# Chronic Inflammatory Arthropathy Preceding Acute Systemic Manifestations of Sarcoidosis: A Possible Overlap of Idiopathic Juvenile Arthritis and Sarcoidosis

**DOI:** 10.1155/2019/6483245

**Published:** 2019-12-07

**Authors:** Matheus Campello Vieira, Priscilla Gomes Tosta, Fernanda Morello Nicole, Lucas Enock V. Roberto, Júlia Guasti P. Vianna, Paulo de Coelho Castro, Rafael Burgomeister Lourenço, Erica Vieira Serrano, Valéria Valim, Weverton Machado Luchi

**Affiliations:** ^1^Federal University of Espírito Santo (UFES), Vitória, ES, Brazil; ^2^Cassiano Antonio de Moraes Hospital, Federal University of Espírito Santo (HUCAM/UFES), Vitória, ES, Brazil; ^3^Radiology Service, Cassiano Antonio de Moraes Hospital, Federal University of Espírito Santo (HUCAM/UFES), Vitória, ES, Brazil; ^4^Rheumatology Service, Cassiano Antonio de Moraes Hospital, Federal University of Espírito Santo (HUCAM/UFES), Vitória, ES, Brazil; ^5^Nephrology Service, Cassiano Antonio de Moraes Hospital, Federal University of Espírito Santo (HUCAM/UFES), Vitória, ES, Brazil

## Abstract

Sarcoidosis is a multisystem disease with unknown etiology, marked by T lymphocytes and macrophages agglomeration, which leads to the formation of noncaseating granulomas in the affected tissues. We describe a case of a 40-year-old black patient referred to our service for evaluation of nephrolithiasis and persistent elevation of plasma creatinine. He reported important weight loss, fever episodes, and abdominal and low back intermittent pain in the past 6 months. The investigation revealed elevated serum calcium level, hepatosplenomegaly, retroperitoneal lymphadenopathy, anemia, thrombocytopenia, and nephrolithiasis. The initial diagnostic hypothesis was lymphoproliferative disease, but the laparoscopic propaedeutic showed multiple white lesions on the liver surface, which biopsy identified as noncaseating granulomas with asteroid corpuscles, suggestive of sarcoidosis. He was treated with corticosteroids with significant improvement in symptoms and in calcium and creatinine levels. Besides, the patient presented a long-term large joints arthropathy, especially on the knees (with bilateral prosthesis), wrists, and ankles, of unknown etiology. We discuss the systemic manifestations of sarcoidosis related to the reported case, as well as the possible overlapping of idiopathic juvenile arthritis with sarcoidosis.

## 1. Introduction

Sarcoidosis is a multisystem disease of unknown etiology, marked by T lymphocytes and macrophages agglomeration, which leads to the formation of noncaseating granulomas in the affected tissues. It mainly affects black patients in the 3^rd^ and 4^th^ decades of life. The course of the disease is unpredictable and may have acute, subacute or chronic presentations, or even be asymptomatic and with spontaneous remission. The most common presentation is the pulmonary involvement with perihilar lymph nodes enlargement and consequent mediastinal widening [1, 2]. Below, we discuss the case of a patient who was referred for investigation of nephrolithiasis and persistent elevation of plasma creatinine, whose medical investigation identified acute sarcoidosis with multiple organs involvement. In addition, the patient had a history of a long-term peripheral arthropathy, which led to the hypothesis of chronic articular sarcoidosis preceding the acute systemic manifestations or the presence of overlapping rheumatoid arthritis (RA) or juvenile idiopathic arthritis (JIA).

## 2. Case Report

A 40-year-old black male patient was referred to the nephrology service to investigate plasma creatinine elevation in the last 6 months. He reported two previous medical evaluations in emergency departments. The first occasion was due to severe abdominal and low back pain with irradiation to hypogastrium, associated with nausea and vomiting. Pancreatic enzymes were elevated and the case was conducted as pancreatitis, but the patient presented no radiological changes. In the second occasion, he had a similar clinical presentation, but with concomitant macroscopic hematuria. Ultrasonography of the urinary tract identified the presence of bilateral nephrolithiasis, microcalculi in the lower third of the right ureter with mild pelvicalyceal dilation and normal-sized kidneys with increased renal parenchymal echogenicity. There was expulsion of the ureteral stone without urological interventions. Because of the persistent elevation of plasma creatinine not justified by nephrolithiasis, the patient was referred for nephrological investigation, but it took some months until the patient got this appointment.

Relevant findings in the physical examination included discolored mucosa +/4, high blood pressure (160/110 mmHg), liver 4 cm from the right costal margin, and palpable spleen and lower limb edema +/4. There were no palpable peripheral lymph nodes. The patient also reported increased urinary volume, sporadic fever and unintentional weight loss around 30 kg over the past 6 months. Past medical history included chronic use of nonsteroidal anti-inflammatory drugs (NSAIDs) and bilateral knee replacement 5 years before presentation, due to a destructive arthropathy of undetermined etiology. He denied previous systemic arterial hypertension, diabetes mellitus, or kidney disease. He was hospitalized for additional investigation.

### 2.1. Additional Investigation

**Figure 1:**
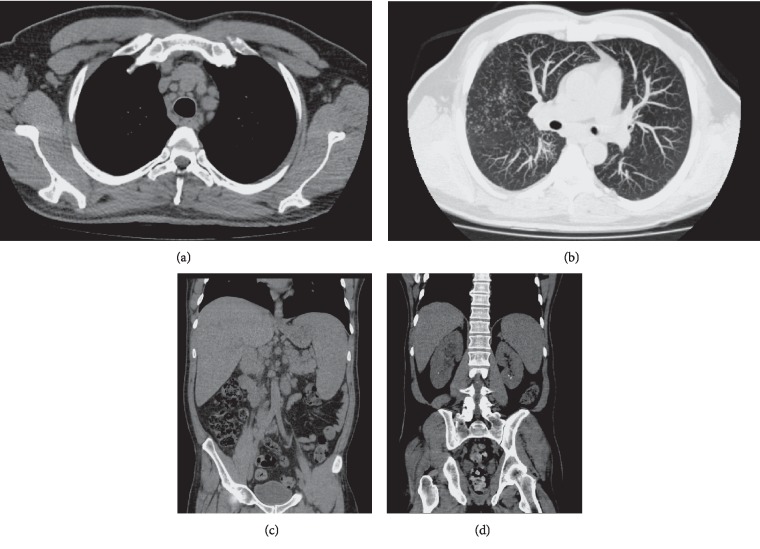
Chest, abdomen, and pelvis computed tomography. (a) Mediastinum with multiple lymph node enlargements in the paratracheal chains. (b) Micronodules in the pulmonary parenchyma with bilateral perilymphatic distribution and enlarged lymph nodes in the pulmonary hilum. (c) Liver and spleen with increased dimensions, and prominent lymph nodes in retroperitoneal and external iliac chains. (d) Kidneys of normal size with nonobstructive calycinal microlithiasis.

Admission laboratory tests revealed several laboratory abnormalities: persistent elevation of plasma creatinine, severe hypercalcemia, anemia, thrombocytopenia, and elevation of pancreatic enzymes and alkaline phosphatase, in addition to changes in urinalysis with nonnephrotic proteinuria, hematuria, leukocyturia, and calcium oxalate crystals (Table 1). In the initial days of hospitalization, diuresis ranged from 4 to 6 L/day. Abdominal computed tomography (CT) showed multiple retroperitoneal and iliac lymphadenopathy, enlarged liver with heterogeneous attenuation, and splenomegaly, as well as multiple nonobstructive renal micro calculi and a normal pancreas (Figure 1). In this scenario, the presence of hypercalcemia associated with a consumptive syndrome and lymphadenopathy suggested the initial hypothesis of a neoplastic condition, more likely a lymphoproliferative disease *a priori*.

**Figure 2:**
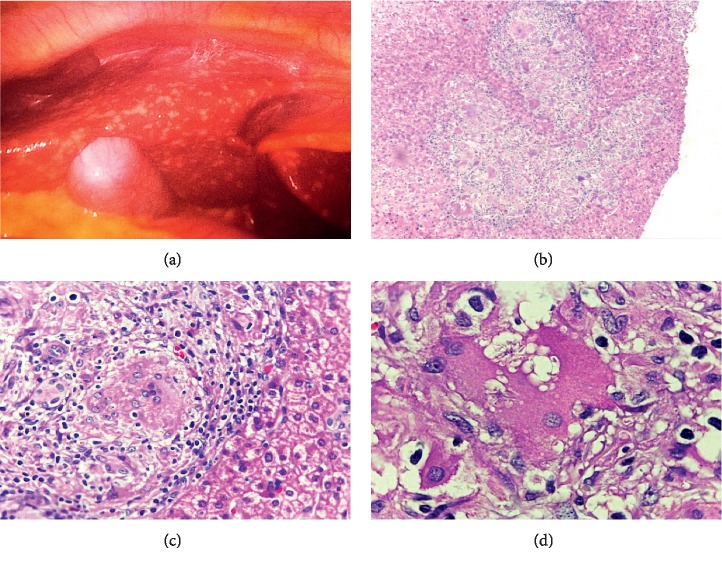
Macroscopic and microscopic appearance of the liver. (a) Multiple white lesions on the liver surface. (b) Well-formed epithelioid granulomas without necrosis (H&E 40x). (c) Epithelioid granuloma with epithelioid macrophages and pooled multinucleated giant cells with interposed lymphocytes (H&E 100x). (d) Detail of multinucleated giant cells with asteroid bodies within the cytoplasm (H&E 1000x), provided by the pathology service of HUCAM-UFES.

The serum parathormone (PTH) level was low, and there was hypercalciuria. Thus, we proceeded the investigation based on differential diagnoses of hypercalcemia with low levels of PTH [3]. There was no history of vitamin D supplementation, and calcidiol level was normal. Unfortunately, we did not have PTH-related peptide (PTHrP) and 1,25(OH)_2_D analysis. Radiographic study of the long bones, skull, and spine did not identify lytic bone lesions. Serum protein electrophoresis identified polyclonal gamma globulin peak, and the myelogram was unchanged. Diagnostic laparoscopy was indicated for retroperitoneal lymph node biopsy; however, intraoperatively it was decided for the performance of liver biopsy since this organ had multiple white lesions (Figure 2(a)). The histopathological study showed epithelioid granulomas without caseous necrosis, with the presence of asteroid bodies, highly suggestive of sarcoidosis (Figures 2(b)–2(d)). Despite the absence of respiratory symptoms, a chest CT scan showed micronodular infiltrates with perilymphatic distribution and mediastinal and hilar lymphadenopathy (Figure 1), corroborating the hypothesis of sarcoidosis. The angiotensin-converting enzyme (ACE) level test was not available in our service. The purified protein-derived (PPD) test for the tuberculosis bacillus was nonreactive (0 mm), and the culture for acid fast bacilli and fungi from the liver biopsy was negative. Ophthalmologic, cardiac, and neurological assessment was unremarkable.

### 2.2. Additional Investigation of Arthropathy

**Figure 3:**
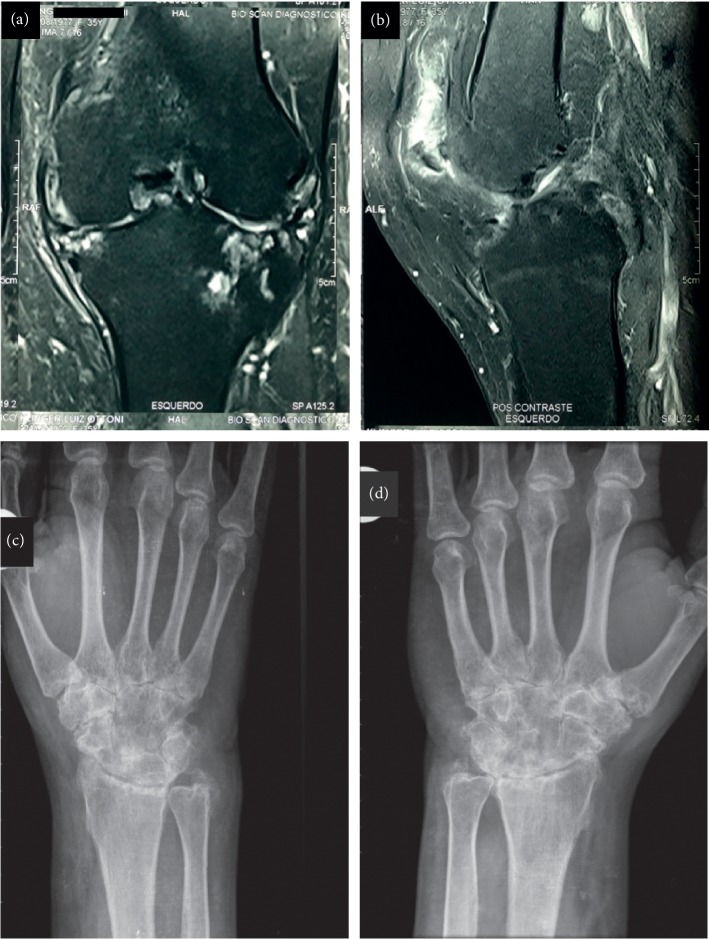
(a and b) Magnetic resonance imaging of the left knee demonstrating an important degenerative process, with bilateral thinning of the articular cartilage and narrowing of the tibiofemoral joint space, bone remodeling, diffuse osteochondral lesions, and intense knee synovitis. (c and d) Wrist radiograph showing the presence of bone demineralization, important diffuse reduction of the interline/joint space, carpal and radial erosions, and diffuse intracarpal ankylosis.

The patient reported recurrent peripheral polyarthralgia since he was 19 years old. The pain started in the wrists and ankles, progressing to elbows, knees, and first metatarsophalangeal joint bilaterally. It was always more severe in the knees, with mixed nociceptive-neuropathic pain characteristic, intermittent, migratory, more often associated with edema and heat, and with relief after use of NSAID. At the age of 34, a magnetic resonance imaging study of the knees was performed, showing an important degenerative process, with bilateral thinning of the articular cartilage and narrowing of the tibiofemoral joint space, bone remodeling, and diffuse osteochondral lesions, as well as intense left knee synovitis (Figures 3(a) and 3(b)). Previous laboratorial tests evidenced high levels of serum uric acid and C-reactive protein and negative rheumatoid factor (RF). Then, he underwent bilateral knee arthroplasty, maintaining a left knee stiffness and loss of range of motion. During the current hospitalization, wrist radiography showed bone demineralization, significant diffuse reduction of joint space, carpal and radial erosions, and diffuse intercarpal ankylosis (Figures 3(c) and 3(d)). The RF dosage was positive. The cyclic citrullinated antipeptide antibody (ACPAs) and the antinuclear antibody (ANA) were negative (Table 1). The synovial fluid presented an intense inflammatory infiltrate with predominance of polymorphonuclears cells (PMN) and absence of crystals. The right wrist synovial biopsy was nonspecific. The Power Doppler Ultrasonography (PDUS) of the wrist showed bilateral carpal joints erosion and intercarpal ankylosis associated with Power Doppler (PD) positive synovitis and tenosynovitis; the metacarpal phalangeal and proximal interphalangeal joints were absent of erosion and synovitis. PDUS of the ankle showed synovial effusion PD-negative of tibiotalar joint and tenosynovitis of tibialis posterior tendon. Diagnostic hypothesis of JIA or sarcoidosis-related arthropathy was made and will be discussed below.

## 3. Discussion

This case describes multiple systemic manifestations of acute sarcoidosis preceded by a chronic inflammatory arthropathy of undefined etiology. We will discuss, separately, the main systemic manifestations of sarcoidosis present in the case, especially the articular and renal, as well as pulmonary, hepatosplenic and pancreatic. We emphasize the articular involvement regarding the differential diagnoses between chronic articular sarcoidosis and overlapping JIA.

### 3.1. Articular Involvement in Sarcoidosis

The reported prevalence of arthropathy in sarcoidosis ranges from 15 to 38%. It usually occurs after other organs involvement, such as lung and eye. Manifestations include nonspecific arthralgia and acute onset arthritis, especially of the ankles (90% cases), followed by involvement of the knees, elbows, wrists, and metacarpophalangeal joints. In 9 to 34% of cases, acute arthritis is associated with erythema nodosum and hilar lymphadenopathy, constituting Löfgren's syndrome. The evolution to chronic articular sarcoidosis is rare (0.2 to 2% of the cases), commonly in the black race and can evolve with Jaccoud arthropathy, articular destruction, soft tissue edema, periarticular osteoporosis, and joint space narrowing, sometimes mimicking RA. Other manifestations might include lytic bone lesions (poor prognosis), dactylitis, and enthesitis. Treatment of the sarcoidosis joint evolvement includes low-dose corticosteroids (10–20 mg/day) or short-term NSAIDs. In cases that culminate with chronicity, disease-modifying antirheumatic drugs such as methotrexate, hydroxychloroquine, azathioprine, sulfasalazine, and colchicine may be used. When there is contraindication or no response to the use of the mentioned drugs, antitumor necrosis factor (anti-TNF) is an alternative [1, 4–8].

In a review article, 14 case reports of sarcoidosis and RA overlap were found from 1980 to 2016 [9–11]. Women were affected in 77% of cases, and in most of them RA preceded sarcoidosis, with the mean age at diagnosis of 35.3 years for RA and 51 years for sarcoidosis. They all presented histopathological evidence of sarcoidosis, mainly by transbronchial biopsy, and none had characteristic osteoarticular biopsy for sarcoidosis. All had positive RF, and when dosed, ACPAs was positive. Considering the high prevalence of RA (1/100) and sarcoidosis (0.4–64/1,000) and the absence of a genetic correlation and a distinct therapeutic response of both diseases, the authors support that the hypothesis of RA overlap with sarcoidosis, instead of a unique pathogenesis, is more likely [11].

Although RA and sarcoidosis overlap is established in the literature, the presence of chronic erosive seronegative polyarthropathy involving wrists and knees, while sparing MCF and IFP associated with negative ACPAs dosage, corroborates in favor to the hypothesis of JIA rather than RA in our case [12]. The positivity of RF during hospitalization may be related to sarcoidosis activity, which is positive in 16.6 to 38% of cases, since previous tests were negative [13, 14]. In addition, RF returned to be negative after 6 months of treatment. Chronic joint involvement, which appeared years before the acute systemic manifestations of sarcoidosis, speaks against the diagnosis of articular sarcoidosis [6]. Considering that synovial fluid in sarcoidosis has poor inflammatory characteristics, mostly at the expense of mononuclear cells or is even noninflammatory, the presence of PMN-rich cellularity suggests JIA diagnosis. Also, synovial biopsy was nonspecific and did not contribute to distinguish the etiology, but the absence of granuloma makes the diagnosis of sarcoidosis less likely [15, 16]. Accordingly, data conglomerate implies that the main diagnostic hypothesis for our patient's articular involvement may be due to JIA. It is possible that joint involvement has started before the age of 16, initially subclinical and becoming symptomatic posteriorly.

### 3.2. Renal Involvement and Hypercalcemia

The incidence and prevalence of renal impairment by sarcoidosis remain unknown, but case series suggest an occurrence in approximately 30 to 50% of patients. In general, it is in the context of other systemic manifestations and is related to lymphomononuclear infiltration into renal tissue or secondarily to hypercalcemia [17]. Around 10–20% of sarcoidosis cases develop hypercalcemia, which is related to the excessive production of calcitriol by the granuloma, consequent to the increased activity of 1a-hydroxylase enzyme by macrophages. In addition to increased intestinal calcium absorption, excessive calcitriol stimulates osteoclast activity and bone resorption. Occasional cases have also described granuloma production of PTHrP in sarcoidosis [18, 19]. Among the manifestations related to the named hypercalcemic nephropathy (hypercalcemia/hypercalciuria) we mention nephrolithiasis, present in about 4% of cases, and in 2.2% it was the first manifestation of sarcoidosis; nephrocalcinosis; renal vasoconstriction; acute tubular necrosis; and nephrogenic diabetes insipidus (NDI). On the other hand, the inflammatory infiltrate will lead to the development of tubulointerstitial nephritis (NTI), a classic manifestation of sarcoidosis in the kidney, which may or not develop with granulomas. Glomerulopathies are uncommon, and the most commonly described were IgA nephropathy and membranous glomerulopathy. Ureteral obstruction is also a rare presentation and could result from lymphadenopathy or retroperitoneal fibrosis. The main clinical manifestations are low back pain, hematuria, polyuria, acute kidney injury, hypertension, and chronic kidney disease (CKD). Urinalysis findings include nonglomerular hematuria (or rarely associated with erythrocyte dysmorphism when there is glomerulopathy), proteinuria (usually <1 g), hypercalciuria, calcium oxalate crystals, granular and leukocyte cylinders, sterile leukocyturia, and eosinophiluria [17, 19–21].

In the reported case, the presence of low back pain and nondysmorphic macroscopic hematuria are compatible with the nephrolithiasis shown on CT. Despite the absence of renal biopsy, findings of proteinuria <1 g/24 h, sterile pyuria, and leukocyte cylinders suggest the diagnosis of NTI, while hypercalciuria and calcium oxalate crystals in urine suggest hypercalcemic nephropathy. Both patterns of kidney injury can cause acute tubular injury, which might be evidenced by the presence of granular cylinders. There were no signs of nephrocalcinosis, ureteral obstruction, or glomerulopathy. In addition to renal injury, the patient had polyuria with hypotonic urine (<250 mOsm/L) and serum sodium >140 mEq/L, suggesting a diagnosis of NDI associated with hypercalcemia/hypercalciuria [20, 22, 23]. Persistence of elevated serum creatinine for more than 3 months initially suggested terminal CKD. However, after corticosteroid therapy, there was a significant improvement in renal function (Table 1). Unfortunately, calcitriol dosage was performed only 3 months after beginning the treatment, which was probably responsible for the finding of normal calcitriol level.

The prednisone treatment recommended for patients with renal dysfunction is 1 mg/kg for 4 weeks with a reduction of 5 mg per week, maintaining a dose of 5–10 mg for 18–24 months. In cases of corticosteroid adverse effects or therapeutic failure, azathioprine, mycophenolate, or an anti-TNF are alternative options [17, 24]. Usually, there is a significant response to corticoid therapy for both NTI and resolution of hypercalcemia. Nevertheless, around 66% of patients develop some degree of CKD, but progression to renal replacement therapy is uncommon [21]. When it occurs, it is often related to hypercalcemic nephropathy than to other forms of renal involvement. In addition to corticosteroid therapy and vigorous hydration, bisphosphonates may also contribute to the treatment of hypercalcemia, but when creatinine clearance is persistently <30 mL/min/1.73 m^2^, preference should be given to the use of denosumab, given the potential nephrotoxic effects of bisphosphonates [25].

### 3.3. Pulmonary Involvement in Sarcoidosis

Sarcoidotic granulomas can be present in any organ, but the lung is the most prevalent affected organ, occurring in more than 90% of patients. The inflammatory process involves alveoli, small bronchi, and small blood vessels. Most patients with pulmonary sarcoidosis are asymptomatic. The most common symptoms, when present, include dyspnea, cough, nonspecific chest discomfort, and wheezing. However, in advanced forms with pulmonary fibrosis, severe hypoxemia and pulmonary hypertension may be present [1, 26].

Although a significant number of patients with pulmonary sarcoidosis have an asymptomatic, nonprogressive, and spontaneously remitting disease, about 50% of the affected patients required systemic corticosteroid therapy [27]. The case reported in this text was asymptomatic in the respiratory aspects, and the chest CT scan only revealed pulmonary infiltrate associated bilateral hilar adenopathy without fibrosis; there was not pulmonary hypertension on echocardiography, and the spirometric parameters were normal. Therefore, it should not be submitted to corticosteroid therapy if only the described pulmonary condition was present; however, the manifestation of the disease in other organs justified the beginning of the treatment.

### 3.4. Hepatosplenic and Pancreatic Involvement in Sarcoidosis

Liver involvement in sarcoidosis is common, ranging from 17 to 90% of cases, but most patients are asymptomatic (60 to 95%), with blacks being the most affected. When symptomatic, signs and symptoms are nonspecific and include fatigue, weight loss, night sweats, and fever. Hepatomegaly is observed on physical examination and imaging in about 21% and 50% of cases, respectively, and abdominal pain in about 15% of patients. Of those with liver involvement, up to 7% may develop cholestatic syndrome, and 3 to 18% may develop portal hypertension, presenting with ascites and esophageal varices. Of patients with portal hypertension, 6 to 26% develop terminal liver cirrhosis and need liver transplant. A noteworthy laboratorial finding is the elevation of alkaline phosphatase, present in more than 90% of cases, reaching levels of 5 to 10 times above the upper limit of the reference. Transaminases, when elevated, are at low levels, and liver function impairment is uncommonly. Hepatic granulomas, represented by nodules, and parenchymal calcifications can be visualized by ultrasound and CT with contrast enhancement, but nuclear magnetic resonance is more sensible [28–30].

Splenic involvement occurs in up to 50% of sarcoidosis patients; however, splenomegaly is detected in only 5–14% of cases on physical examination. Abdominal pain in the left upper quadrant is uncommon, and most patients are asymptomatic, with nonspecific symptoms, similar to hepatic involvement. Severe splenomegaly associated with hypersplenism and pancytopenia occur in 20% of those affected. Splenic rupture is a rarely described event [30].

Pancreatic involvement is also uncommon, occurring in 1 to 5% of patients. There is often elevation of pancreatic enzymes without symptoms, but symptomatic cases may present pain, jaundice, and anorexia. Pancreatitis might be due to granulomatous involvement of the pancreas or secondary to hypercalcemia [31, 32].

In the reported case, in agreement with most cases described in the literature, the patient had unspecific symptoms of hepatosplenic involvement associated with increased alkaline phosphatase, although he had hepatosplenomegaly [30]. Considering that the myelogram was normal, the present anemia and thrombocytopenia are probably related to hypersplenism. A presumptive diagnosis of pancreatitis was given to the patient during his first visit to the emergency department due to abdominal pain and elevation of amylase and lipase, even though pancreatic abnormalities on CT were absent. However, pancreatic enzymes remained elevated throughout the course of the disease, and the diagnosis of nephrolithiasis was made after a similar new episode of abdominal pain. Therefore, it is likely that the first episode of abdominal pain was also caused by an acute renal colic rather than acute pancreatitis and that pancreatic involvement was due to asymptomatic elevation of pancreatic enzymes (Table 1).

### 3.5. Case Follow-Up

Hypercalcemia was initially managed with vigorous hydration, despite renal injury, since the patient had polyuria due to NDI. Intravenous bisphosphonate was added for hypercalcemic control because the early diagnostic hypothesis was some malignant conditions, where the production of PTHrP is the main mechanism. There was partial improvement of hypercalcemia and renal function (Table 1). Then, after the established diagnosis of sarcoidosis, corticosteroid therapy was instituted (oral prednisone 1 mg/kg in the first month, with progressive reduction). The patient evolved with clinical and laboratory improvement after the first month of treatment, with normalization of serum calcium and resolution of urinalysis alterations (Table 1). Joint pain also had partial improvement with corticosteroid therapy. The patient progressively recovered his normal weight, and serum creatinine levels stabilized around 1.5 mg/dL after the twelve months of treatment. At this time, prednisone withdrawal was started, and the treatment of the supposed overlapping JIA was initiated due to the severity of the arthropathy. Because of the significant renal injury, methotrexate was not chosen as the first drug due to the potential nephrotoxic effect, and an anti-TNF corticosteroid-sparing agent was added to the therapy regimen, which would also be effective in the treatment of sarcoidosis.

## Figures and Tables

**Table 1:** Laboratorial data follow-up.

	Six months before	At admission	3 months after stating treatment	Twelve months after starting treatment	Reference values
Blood					
Hemoglobin (g/dL)	10.8	11.6	11	12.7	12–15
White blood cells (mm^3^)	9040	11.600	13.900	10.750	4.000–12.000
Platelet (mm³)	262.000	50.000	193.000	289.000	150.000–400.000
Creatinine (mg/dL)	2.3	5.9	1.81	1.5	0.4–1.3
Urea (mg/dL)	60	149	110	40	15–45
Sodium (mEq/L)	141	144	143	140	135–145
Potassium (mEq/L)	3.7	3.39	4.7	4.9	3.9–5.0
Calcium (mg/dL)	—	17.4	9.8	9.9	8.8–11.0
Phosphorus (mg/dL)	—	6.6	3.4	3.3	2.5–4.8
Intact PTH (pg/mL)	—	13	—	85	12–72
25-OH-vitamin D (ng/mL)	—	12.1	11.7	19.7	30–100
1.25-OH-vitamin D (pg/mL)	—	—	9.5	—	15.2–90.1
Uric acid (mg/dL)	—	9	7.7	7.5	2.5–7.0
Albumin (g/dL)	—	4.1	4.0	—	3.5–5.2
Alkaline phosphatase (U/L)	198	316	—	100	40–129
Gama GT (U/L)	32	36	—	24	8–61
TGO/TGP (U/L)	13/19	12/13	—	22/34	10–50
Amylase (U/L)	328	142	—	65	28–100
Lipase (U/L)	546	115	—	19	15–60
C-reactive protein (mg/L)	—	47	0.6	—	<5
Rheumatoid factor (UI/mL)	—	43	—	<20	<20
ACPAs (U/mL)	—	9.9	—	—	<20
Antinuclear antibodies	—	Negative	—	—	Negative
Urine					
Spot					
pH	6	6.5	—	5	4.5–8
Density	1.015	1.010	—	1.013	1.005–1.035
Proteinuria	+	+	—	Negative	Negative
Red blood cells (/hpf)	18	30	—	1	<5
Leukocyturia (/hpf)	25	7	—	1	<5
Crystals	Calcium oxalate +	Calcium oxalate +++	—	Negative	Negative
Casts	Granular ++	Granular + leucocyte+	—	Negative	Negative
24 hour					
Urinary volume (mL)		4,500		3,200	<3,000
Albuminuria (mg/24 h)	—	863	—	60	<30
Proteinuria (mg/24 h)	—	2.330	—	200	<150
Calcium (mg/24 h)	—	373	—	33	100–300
Osmolarity (mOsm/L)	—	220	—	—	50–1200
